# Advancing Care in Lupus Nephritis: Expert Perspectives on Current Practices and Future Directions in Italy

**DOI:** 10.3390/jcm15103570

**Published:** 2026-05-07

**Authors:** Andrea Doria, Franco Franceschini, Loreto Gesualdo, Gabriella Moroni, Marta Mosca, Renato Alberto Sinico, Dario Roccatello

**Affiliations:** 1 Rheumatology Unit, Department of Medicine (DIMED), University of Padua, 35128 Padova, Italy; 2 Rheumatology and Clinical Immunology Unit, Department of Clinical and Experimental Sciences, ERN ReCONNET Center, ASST Spedali Civili, University of Brescia, 25121 Brescia, Italy; franco.franceschini1@gmail.com; 3 Department of Precision and Regenerative Medicine and Ionian Area (DiMePre-J), University of Bari, 70124 Bari, Italy; 4 Department of Biomedical Sciences, Humanitas University, 20072 Pieve Emanuele, Italy; 5 Nephrology and Dialysis Unit, IRCCS Humanitas Research Hospital, 20089 Rozzano, Italy; renato.sinico@humanitas.it; 6 Rheumatology Unit, Department of Clinical and Experimental Medicine, University of Pisa, 56126 Pisa, Italy; 7 University Center of Excellence on Nephrology, Rheumatology and Rare Diseases, Nephrology and Dialysis Unit-CMID, ERKnet, ERNReCONNET and RITA-ERN, S. Giovanni Bosco Hospital, University of Turin, 10154 Turin, Italy

**Keywords:** lupus erythematosus, lupus nephritis, immunosuppressive agents, intensified B-cell depletion therapy, obinutuzumab, anifrolumab

## Abstract

**Background:** Lupus nephritis (LN), a major complication of systemic lupus erythematosus, remains a key determinant of morbidity and mortality despite therapeutic progress. **Objective:** An expert report aims to present multidisciplinary insights from leading Italian centers on current LN management and future perspectives. **Methods:** Seven specialists—including nephrologists and rheumatologists with expertise in lupus nephritis—addressed key aspects of LN management, including treatment goals, available therapeutic options, treatment selection across disease stages, and emerging strategies. **Results:** The authors highlight evolving treatment paradigms—shifting from traditional induction–maintenance strategies to early combination regimens integrating biologics such as belimumab and calcineurin inhibitors like voclosporin—aligned with updated EULAR and KDIGO guidelines. Regional experiences underscore individualized therapy tailored by histology, disease activity, and patient profile. Multidisciplinary collaboration between nephrologists and rheumatologists, equitable access to innovative therapies, and integration of registries and digital records are identified as priorities. **Conclusions:** Emerging agents, including obinutuzumab, anifrolumab, and CAR-T cell therapies, represent promising advances toward steroid-sparing and potentially curative strategies. Continued research, biomarker development, and application of artificial intelligence are pivotal to optimizing outcomes and achieving personalized LN management.

## 1. Introduction

Lupus nephritis (LN), a frequent manifestation of systemic lupus erythematosus (SLE), significantly predicts reduced health-related quality of life, higher mortality risk, and economic burden [1,2]. Lupus nephritis affects approximately 40% of SLE patients, with over 10% progressing to end-stage renal disease; it can be present at the time of SLE diagnosis or appear later, mostly within five years [3]. Male sex and LN at SLE onset may lead to more severe kidney outcomes [3,4]. Older patients also show significantly worse outcomes due to advanced chronicity and systemic impairment [5].

In SLE patients, assessing kidney function and performing renal biopsy is crucial to diagnose LN, guide treatment, and prevent irreversible renal damage [6]. The Nephrology/Renal Pathology International Society classification is the gold standard for characterizing LN via renal biopsy, guiding therapy [7]. LN is divided into six histological classes. Class I and II refer to minimal and proliferative mesangial glomerulonephritis, respectively. Class III denotes focal glomerulonephritis, and class IV indicates diffuse glomerulonephritis. Class V applies to membranous glomerulonephritis, and class VI refers to advanced sclerosing glomerulonephritis [8]. The most common glomerular pathology in LN is secondary to immune complexes deposition; their presence and location can be confirmed by ultrastructural analysis using electron microscopy [8]. Renal function may be compromised by glomerular, tubulointerstitial, and vascular lesions, which must be reported due to their prognostic significance [6,7].

A crucial role in immune responses that characterize SLE pathogenesis is played by B cells and plasma cells secreting autoantibodies. Therefore, they represent the main targets in SLE and LN therapeutic approaches, with significant changes occurring over the decades.

An Italian study involving 499 cases treated between 1970 and 2016 showed a low survival rate prior to 1985 [9]. After cyclophosphamide (CYC) was introduced as a treatment for LN, there was a significant improvement in survival rates. Further improvements were then observed with the reduction in the steroid dose, optimization of CYC-protocols, and introduction of mycophenolate mofetil (MMF) [9].

Despite therapeutic advances, renal outcomes remain suboptimal; between 2002 and 2016, 58.5% patients achieved remission, 32.1% partial remission, and 9.4% progressed to chronic kidney disease, ESRD, or death [3,9].

The standard of care was mycophenolate mofetil (MMF) or cyclophosphamide administered intravenously (CYC), followed by pulses of methylprednisolone and a gradual reduction in oral prednisone [10].

Subsequent studies [11] introduced updated treatment strategies, validated therapeutic goals, alternative glucocorticoid-sparing regimens [12], and combination therapies using calcineurin inhibitors (voclosporin) [13], belimumab (IgG1λ monoclonal antibody) [14], and emerging agents [15,16,17].

In 2019, the European Alliance of Associations for Rheumatology (EULAR)/European Renal Association/European Dialysis Transplantation Association (ERA-EDTA) updated its recommendations to encompass the evaluation of renal and extra-renal disease activity, refined therapeutic targets, optimized treatment protocols, and ESRD management.

Despite these advances, LN still poses significant therapeutic challenges and unmet clinical needs.

The management of SLE and kidney involvement should follow the same recommendations as for the main condition, including hydroxychloroquine [11]. Treatment regimens for LN include intravenous methylprednisolone followed by oral glucocorticoid in combination, with either oral mycophenolate mofetil or low-dose intravenous cyclophosphamide, or combination therapies recommended by KDIGO 2024 Clinical Practice Guideline for the Management of Lupus Nephritis, published in 2024 [18] and the very recent EULAR/ERA-EDTA guidelines for patients with active NL and poor prognostic factors [19]. Both converge on early biopsy, treat-to-target principles, long maintenance therapy, and steroid minimization (Table 1).

KDIGO 2024 [18] provides the most granular renal-specific guidance, especially regarding definitions of response, timelines, and biopsy-driven decisions. EULAR 2025 [19] therapy introduces a more aggressive combination-therapy paradigm, explicitly incorporating belimumab, voclosporin, tacrolimus, and obinutuzumab, and sets a clear proteinuria target at 12 months. The EULAR 2025 [19] recommendations do not specifically mention rituximab, which remains widely used worldwide, being the most cost-effective immunomodulatory agent in these diseases. Rituximab may be considered for patients with persistent disease activity or inadequate response to initial standard-of-care therapy according to KDIGO 2024 guidelines and should certainly be considered among the combination regimens that use “B-depleting” agents in refractory forms mentioned by EULAR 2025 recommendations [18,19].

In March 2025, the authors, comprising nephrologists and rheumatologists, convened to discuss LN management challenges, share multidisciplinary strategies, review practices at Italian excellence centers, and evaluate future therapeutic perspectives in this field. Indeed, following the revision of Article V of the Constitution of the Italian Republic [20], Italy has strengthened its commitment to local autonomy through full implementation of administrative decentralization. As a result, the organization of public healthcare varies markedly across regions, providing diverse perspectives and clinical approaches, particularly in areas where therapeutic guidelines remain controversial [21]. A distinctive national feature is the absence of strict treatment limitations, alongside a strong emphasis on the use of biosimilars—especially in northern regions—which reduces the cost of biotechnological therapies and improves their accessibility and cost-effectiveness. This heterogeneity has attracted international scientific interest, as it enables the evaluation of systematically documented alternative approaches in the literature.

Seven specialists from across Italy, including nephrologists and rheumatologists with expertise in lupus nephritis, were invited by the corresponding author to participate in this project, forming a group with broad clinical experience to exchange real-world insights on disease management. Actually, the experts were selected based on their scientific contributions to the field of lupus nephritis, as well as their leadership roles in hub centers across Italy. This approach also enabled the collection of information from the surrounding spoke centers within their networks, thereby ensuring broad coverage of the national territory. The group is self-referential and does not represent any European or national scientific society.

This expert report aims to summarize multidisciplinary perspectives from leading Italian centers on current and emerging strategies for the management of SLE and kidney involvement, in the hope that the authors’ experience may contribute to improving clinical practice beyond national borders.

## 2. Treatment Approaches for LN in Italy

The authors agreed that management of SLE with kidney involvement has transformed, shifting from conventional induction-maintenance approaches to early implementation of combination therapeutic strategies.

In 2011, belimumab [22] became the first biologic drug approved for SLE in over 50 years, and in June 2022, it was authorized and reimbursed by the Italian Medicines Agency, in patients aged 5 years and over, for active LN in combination with immunosuppressants.

Belimumab is a human IgG1λ monoclonal antibody that binds specifically to the soluble human B lymphocyte stimulator (BLyS) protein, also known as B-cell activating factor (BAFF). It binds to BLyS, thereby inhibiting its interaction with the three receptors (BAFF-R, TACI, and BCMA) on B cells. This reduces the proliferation and survival of B cells, particularly autoreactive B cells, as well as the differentiation of B cells into immunoglobulin-producing plasma cells.

Following this, in June 2023, voclosporin [23] was approved for adult patients with active class III, IV, or V LN—including mixed III/V and IV/V—in combination with MMF. Voclosporin is an immunosuppressant that inhibits calcineurin, a calcium/calmodulin-dependent phosphatase whose activity is required for the induction of T-cell lymphokine production and proliferation.

The prescription and dispensing of both drugs are confined to specialists at hospital centers officially designated by the administrative authorities of each Italian region.

Rituximab, a chimeric monoclonal antibody that targets the CD20 antigen receptor on B cells, has also been proposed as an alternative treatment for LN. The evidence supports the fundamental role of B cells in producing autoantibodies and in activating and modulating dendritic cell and T cell functions, including tolerance, the induction of autoreactive memory T cells, and the activation of Th1 and Th17 cells [24].

The authors report that, based on their experience with centers across northern, central, and southern Italy, the following distinctions can be highlighted. In Bari (southern Italy), nephrologists reported following the classic LN approach per 2024 KDIGO guidelines, favoring MMF and CYC, especially for fibrinoid necrosis and severe acute renal failure cases. Rheumatologists in Pisa (central Italy), Brescia (north Italy), and Padua (north-east Italy) considered combination therapy the best treatment option for LN due to its clinical benefits and tolerability. Combination therapy with belimumab or voclosporin is considered standard care, providing maximal efficacy during induction, with selection guided by histology and clinical variables [25]. The Humanitas center (northern Italy, Nephrology) favors combination therapy but acknowledges that traditional dual therapy is effective for patients with class III LN, proteinuria ≤ 1 g/g, and a low activity index. CYC is reserved for clinically or histologically severe cases or patients with limited compliance. The Turin Center, in northwestern Italy, Nephrology, has a robust experience using the intensified B-cell depletion protocol—six rituximab (375 mg/m^2^) and two low-dose CYC (10 mg/kg) administrations—for proliferative and class V LN without maintenance treatment [17]; this is also given as a front-line regimen because of its cost-effectiveness. It should be emphasized that with the advent of biosimilars, the cost of rituximab can be negotiated in Italy at less than €100 per 375 mg/m^2^ infusion [26].

B cells, plasma blasts, and plasma cells are key sources of autoantibodies in SLE, and this therapeutic protocol efficiently targets B cells.

Although confirmation from a large-scale randomized controlled trial is still awaited [27], the intensified B-cell depletion protocol has been shown to be more effective than standard care, inducing long-lasting remission in patients with active LN [28]. In a prospective study, remission was maintained with minimal prednisone doses starting at the end of the third month after the induction regimen, thwarting the need for immunosuppressive maintenance therapy [17]. This led to significant corticosteroid sparing and a substantial reduction in the risk of immunosuppression.

All the authors agreed that, given voclosporin’s recent introduction in Italy, experience with its use remains limited across all centers.

## 3. Goals of the Therapeutic Strategy in NL

The authors agreed that LN therapy should focus on three core objectives: improving response, preventing flares, and minimizing steroids.

To maintain long-term improvement, it is essential to promptly treat the patient, considering that each disease flare-up results in a loss of nephrons, which can lead to chronic damage. Malvar et al. examined changes in renal histology over time in 110 patients with LN undergoing immunosuppressive treatment [29]. Early intervention and remission within the first year were linked to improved long-term survival [29]. Accordingly, achieving a deep response within 12 months and maintaining clinical remission are crucial to prevent renal flares and disease progression, highlighting immunosuppressive therapy’s initial role in avoiding irreversible chronic kidney damage [30].

The updated 2025 EULAR recommendations provide an evidence- and expert-based consensus on the management of systemic lupus erythematosus (SLE) with kidney involvement. Treatment should aim to preserve or improve kidney function within three months, alongside reducing proteinuria by at least 25% within three months, by 50% within six months, and achieving a urine protein-to-creatinine ratio target of less than 0.7 g/g within 12 months, with the aim of achieving the lowest possible ratio thereafter [19].

The authors concurred that, in accordance with the assessment of the nephrologist and rheumatologist, a re-biopsy may be warranted during LN clinical exacerbation, refractoriness to therapy, or before treatment discontinuation following remission, as repeat kidney biopsies—especially when biochemical markers are inconclusive—offer valuable insights for managing LN and predicting long-term prognosis [31].

The duration of immunosuppressive therapy is also a topic of debate. A recent study showed that resolving inflammatory kidney injury in LN often requires years of treatment, emphasizing the need for well-tolerated maintenance immunosuppression [32]. Thus, the traditional induction–maintenance protocol can be replaced by a personalized, flexible approach targeting four goals [33]: (a) achieving LN clinical remission; (b) preserving renal function [32]; (c) reducing SLE activity by decreasing immune complex generation and attaining immunological remission; and (d) preventing future LN relapses [30].

Of course, regardless of the protocol chosen, limiting the use of glucocorticoids remains crucial. LN treatment regimens have substantial side effects due to prolonged immunosuppression. The prolonged use of glucocorticoids poses a significant challenge, despite their pivotal role in managing SLE. Glucocorticoids have many adverse effects, including osteoporotic fractures, avascular necrosis, diabetes mellitus, cataracts, glaucoma, coronary heart disease, cognitive impairment, and increased mortality [34,35].

Therefore, the tendency is to use the lowest possible dose of steroids and employ strategies to prevent or limit glucocorticoid-induced side effects [12,36]. The most widely endorsed treatment regimen consists of intravenous pulse methylprednisolone, followed by oral glucocorticoids gradually reduced to ≤5 mg/day of prednisone equivalents over a period of 4 to 6 months [11].

Early immunosuppression is essential in LN, allowing use of the lowest effective steroid dose to minimize glucocorticoid-related adverse effects while effectively controlling disease [12].

Emerging therapies, primarily CAR-T cell therapy, aim to reset aberrant autoimmunity through the deep depletion of B cells as a potential strategy for achieving sustained drug-free remission [37]. Concerns have arisen regarding the actual feasibility of achieving these results in every case in a cost-effective way [38].

## 4. Multidisciplinary Management and Equity in Healthcare Access

The board stresses strengthening rheumatologist–nephrologist collaboration for multidisciplinary SLE and LN care, with standardized guidelines and integrated digital records to reduce disparities and ensure equitable access [39]. Mapping patient journeys through clinical pathways helps resolve bottlenecks and ensures timely access to diagnostics and innovative therapies across all centers nationwide [37].

The authors highlighted that LN-specific registries integrated with electronic health records can standardize documentation and track disease activity, disease manifestations, and treatments. Additionally, patient education, empowerment, and emotional support are essential due to SLE’s chronic nature and therapy-related burdens [40].

Finally, cross-border cooperation among specialized healthcare services, clinical studies, and research may accelerate the delivery of more appropriate care for patients.

In summary, Italy employs both treatment regimens that are strongly aligned with international guidelines and unconventional therapeutic strategies, including first-line treatments developed based on long-term experience and cost-effectiveness parameters, which are favored by regional regulations that are relatively open to off-label solutions.

## 5. Views on Future Perspectives

### 5.1. Emerging Therapies

Obinutuzumab, a humanized, glycoengineered type II anti-CD20 IgG1 monoclonal antibody, has shown efficacy and safety in LN. In the REGENCY phase 3 randomized controlled trial, which included 271 adults with active LN, obinutuzumab plus standard therapy was more effective than standard therapy alone in achieving a complete renal response [41]. Considering the trial’s primary endpoint, complete renal response, 46.4% of patients randomized to obinutuzumab treatment achieved complete renal response at week 76, compared with 33.1% of those randomized to placebo. This allowed for a reduction in the steroid dosage [41].

Obinutuzumab showed greater improvements in other renal response measures, serologies, estimated glomerular filtration rate, and proteinuria [41,42].

Anifrolumab, a human monoclonal antibody targeting the type I interferon receptor, reduces SLE disease activity. A phase 3 study evaluated its efficacy and safety in active proliferative LN. By blocking IFNAR1, it broadly dampens interferon-driven inflammation, whereas obinutuzumab selectively depletes CD20^+^ B cells, targeting a key pathogenic population in LN through cytotoxicity and direct cell death [43].

Daratumumab monotherapy in resistant, biopsy-proven LN has been extensively studied in Turin (northwestern Italy). It was shown to induce sustained clinical response in refractory patients at 12 months, with immuno-monitoring revealing suppressed interferon pathway activation and transcriptomic shifts in T-cell phenotypes [16].

No unexpected safety signals were observed with these molecules; consistent with the known safety profile of monoclonal antibody therapies, adverse effects included infusion reactions, flu-like symptoms, gastrointestinal disturbances, and an increased risk of infection [16,41,43].

Antibodies targeting B-cell maturation antigen (BCMA) and CD19 are emerging as potential treatments for SLE [44]. The most promising are bispecific antibodies, which include two targets in their structure; for example, CD3 on T-cells and CD19 (or CD20) on B cells. Ligation of CD3 activates a T cell, which then induces direct B-cell killing. It is expected that this mechanism will be much more effective at eliminating tissue-resident B cells than traditional monoclonal antibodies, which are highly dependent on antibody-dependent cytotoxicity. A bispecific CD20/CD3 T-cell-engaging antibody, mosunetuzumab, is under development [45,46].

These new approaches are entering the therapeutic arsenal of the major Italian centers, and the results of these new strategies will soon be delivered to the literature.

For SLE extremely refractory to conventional therapy, emerging therapies include CD19-directed CAR-T autologous cell treatments [38]. In 2025, the ADI-001 trial (NCT06375993) received fast-track designation from the FDA for the treatment of refractory systemic lupus erythematosus with extrarenal involvement [47]. This phase 1 trial uses allogeneic (off-the-shelf) gamma delta CAR-T cells to target B cells in autoimmune diseases, including a lupus nephritis (LN)-specific cohort. Other CAR-T cell trials (NCT06152172 [48], NCT06839976 [49], NCT06189157 [50], NCT06121297 [51]) are currently underway.

Clinical trials of new Janus kinase inhibitors (JAKi) in LN patients are also ongoing: Filgotinib (NCT03285711) [52], baricitinib (NC043143747) [53], and deucravacitinib [54]. However, besides the tricky definition of refractoriness in SLE, which probably needs to include both SOC and biologic agents, the cost and complexity of these treatments will probably need a unified evidence base to be justified. Furthermore, a curative treatment that poses a significant safety risk may be less appealing than a safer therapy that may not be entirely curative.

### 5.2. Biomarkers

The authors agree that scientifically validated, non-invasive biomarkers integrated with renal histology could enhance LN management; however, biomarker development is hindered by LN’s complex pathogenesis, ethnic and geographic variability, and lack of standardized treatment protocols [55].

### 5.3. Artificial Intelligence

Machine learning models, at the forefront of innovation for SLE patients, hold great potential to improve diagnosis, prognosis, and treatment personalization, but further research is required to clarify their full capabilities and limitations [56].

Finally, we present Table 2, which summarizes the current therapies for lupus nephritis. The authors anticipate that the prompt regulatory approval of new, promising molecules may contribute to current treatment strategies.

## 6. Conclusions

The variable nature of SLE makes it difficult to standardize patient treatment. Current therapeutic options, new biologic therapies, and combination therapies of biologics with standard immunosuppressive drugs were evaluated by the authors, who are opinion leaders from Italian centers of excellence in the treatment of LN.

They emphasized the importance of implementing a targeted treatment strategy that aims to reduce or discontinue glucocorticosteroid therapy while achieving stable disease control. The advent of targeted biologic therapies has led to the approval of the biologic drugs belimumab and anifrolumab and a more potent and manageable calcineurin inhibitor (voclosporin).

The convergence of KDIGO 2024 and EULAR 2025 pushes Italy toward a more structured, biopsy-anchored, combination-therapy model for LN [18,19]. The real differentiator will be regional capacity to operationalize monitoring, access to new drugs, and steroid-sparing protocols within existing governance frameworks.

Treat-to-target timelines (3–6–12 months) demand standardized monitoring protocols for proteinuria, eGFR, and immunological markers, with shared dashboards between specialties. Biopsy-driven decision making reinforces the need for timely access to nephropathology services, which remains uneven across Italian regions. Steroid minimization strategies require cultural and organizational change, especially in centers historically reliant on higher-dose regimens. Shift toward combination therapy requires alignment between rheumatologists, nephrologists, and pharmacists to ensure consistent adoption across regions. The uptake of belimumab, voclosporin, and obinutuzumab depends on the Italian Medicines Agency positioning, regional formularies, and hospital budget; regions with limited access to biologics or delayed procurement risk heterogeneous implementation of EULAR 2025 recommendations [19].

Care pathway integration in LN management is now a strategic point in Italy for policy and governance discussion to ensure access and equity.

## Figures and Tables

**Table 1:** KDIGO 2024 [18] vs. EULAR 2025 [19] in LN management.

	KDIGO 2024 [18]	EULAR 2025 [19]
Document scope	Kidney-specific guideline with GRADE methodology; detailed algorithms for diagnosis, induction, maintenance, and relapse.	SLE guideline with dedicated renal section; 4 overarching principles and 13 recommendations for kidney involvement.
Renal biopsy	Mandatory for significant proteinuria, active sediment, or unexplained eGFR decline; central to classification and treatment decisions.	Required in all patients with suspected renal involvement; emphasized as essential for diagnosis and management.
Treatment targets	Structured definitions of partial/complete response with timelines (3, 6, 12 months).	Explicit target: urine protein-to-creatinine ratio (UPCR) < 700 mg/g at 12 months and as low as possible thereafter.
Induction therapy (Class III/IV ± V)	Glucocorticoids + one of the following: mycophenolate mofetil (MMF) or mycophenolic acid analogs; low-dose intravenous cyclophosphamide; MMF + calcineurin inhibitor (voclosporin or tacrolimus) when eGFR is adequate; MMF or cyclophosphamide + belimumab.	Combination therapy recommended: MMF or low-dose cyclophosphamide + belimumab; MMF + calcineurin inhibitor (voclosporin or tacrolimus); MMF + obinutuzumab.
Class V	Combined immunosuppressive treatment with glucocorticoid and one other agent (e.g., mycophenolic acid analogs, cyclophosphamide, calcineurin inhibitor, rituximab, azathioprine).	
Biologics and CNI	Belimumab and voclosporin as combination options; role modulated by eGFR and risk profile.	Belimumab, voclosporin/tacrolimus, and obinutuzumab explicitly recommended as part of combination regimens.
Unsatisfactory response	No improvement or worsening despite treatment for 3–4 weeks	Lack of optimization or improvement in kidney function, and/or failure to achieve a reduction in proteinuria of at least 25% by 3 months.
Maintenance therapy	MMF preferred; azathioprine as an alternative; minimum duration ≥ 3 years with gradual tapering based on response and biopsy findings.	Continue therapy for ≥3 years after achieving response; consider tapering or discontinuation only in sustained remission, sometimes after repeat biopsy.
Management of refractory LN	Considers biologic therapy such as belimumab; allows use of calcineurin inhibitor–based combinations when kidney function permits; acknowledges that rituximab may be considered in selected refractory cases based on clinician judgment and available evidence.	Emphasizes combination therapy such as mycophenolate mofetil plus belimumab, mycophenolate mofetil plus calcineurin inhibitor (voclosporin or tacrolimus), or mycophenolate mofetil plus obinutuzumab. Explicitly notes that rituximab may be used in refractory disease when other options are insufficient or contraindicated.
Repeat biopsy	Recommended for suboptimal response, unexpected deterioration, or before major therapeutic changes.	Suggested in cases of clinical uncertainty, including before tapering immunosuppression in long-term remission.
Glucocorticoids	Strong emphasis on steroid minimization with lower starting doses and rapid taper.	Aim for ≤5 mg/day prednisone equivalent and discontinue when possible; short IV pulses for moderate–severe disease.
Nephroprotection	Renin–angiotensin–aldosterone system blockade; blood pressure control; cardiovascular risk reduction; infection and bone health management.	Same measures, with the addition of sodium–glucose cotransporter 2 (SGLT2) inhibitors in selected stable patients.

**Table 2:** Current therapeutic options in lupus nephritis.

	Belimumab	Voclosporin	Obinutuzumab	Anifrolumab	Rituximab
Drug class	Monoclonal antibody against B lymphocyte stimulator (BLyS/BAFF inhibitor)	Next-generation calcineurin inhibitor	Type II anti-CD20 monoclonal antibody	Monoclonal antibody blocking type I interferon receptor (IFNAR1)	Type I anti-CD20 monoclonal antibody
Primary immunologic target	Reduces B-cell survival and autoantibody production	Inhibits calcineurin → reduces T-cell activation	Depletes CD20+ B cells with enhanced antibody-dependent cytotoxicity	Blocks type I interferon signaling, a key SLE inflammatory pathway	Depletes CD20-positive B cells (classical B-cell depletion)
Key clinical trials in LN	BLISS-LN [57]	AURORA-1 [58] and AURORA-2 [59]	REGENCY [41] NOBILITY [42]	TULIP-LN (phase II; did not meet primary endpoint) TULIP SC [60]	LUNAR (phase III; primary endpoint not met) [61] RITUXILUP trial (high response rates) [62] and multiple obsevational studies [63]
Role in guidelines	Included in combination regimens in KDIGO 2024 and EULAR 2025 for proliferative LN [18,19]	Included in KDIGO 2024 and EULAR 2025 as part of combination regimens [18,19]	Included in EULAR 2025 as an option in combination therapy [19]	Mentioned in SLE guidelines; not recommended for LN due to insufficient evidence	Mentioned in KDIGO 2024 as a possible option in refractory lupus nephritis [18]
Administration	Intravenous or subcutaneous	Oral	Intravenous	Intravenous Subcutaneous [60]	Intravenous
Safety profile (general)	Generally well tolerated; risk of infections, depression, infusion reactions	Requires monitoring for nephrotoxicity, hypertension, drug–drug interactions	Infusion reactions, infections, hypogammaglobulinemia	Herpes zoster risk, infections, infusion reactions	Infusion reactions, infections, hypogammaglobulinemia,
Regulatory status in Italy	Authorized in Italy for hospital use as an add-on therapy for SLE, including LN.	Authorized in Italy in combination with MMF for active class III, IV, or V LN (including mixed classes III/V and IV/V).	Not yet approved for LN	Authorized for moderate to severe SLE despite standard therapy. Not yet approved for LN	Not yet approved for LN Off-label use permitted for refractory LN in specialized centers.

## Data Availability

Data available from the corresponding author upon reasonable request.
